# Electronic Structure Engineering of Highly‐Scalable Earth‐Abundant Multi‐Synergized Electrocatalyst for Exceptional Overall Water Splitting in Neutral Medium

**DOI:** 10.1002/advs.202203678

**Published:** 2022-11-11

**Authors:** Gaurav Bahuguna, Adam Cohen, Boris Filanovsky, Fernando Patolsky

**Affiliations:** ^1^ School of Chemistry Faculty of Exact Sciences Tel Aviv University Tel Aviv 69978 Israel; ^2^ Department of Materials Science and Engineering The Iby and Aladar Fleischman Faculty of Engineering Tel Aviv University Tel Aviv 69978 Israel

**Keywords:** bifunctional catalysts, hydrogen production, neutral water splitting, nickel sulfides

## Abstract

Efficient neutral water splitting may represent in future a sustainable solution to unconstrained energy requirements, but yet necessitates the development of innovative avenues for achieving the currently unmet required performances. Herein, a novel paradigm based on the combination of electronic structure engineering and surface morphology tuning of earth‐abundant 3D‐hierarchical binder‐free electrocatalysts is demonstrated, via a scalable single‐step thermal transformation of nickel substrates under sulfur environment. A temporal‐evolution of the resulting 3D‐nanostructured substrates is performed for the intentional enhancement of non‐abundant highly‐catalytic Ni^3+^ and *p*S_n_^2−^ species on the catalyst surface, concomitantly accompanied with densification of the hierarchical catalyst morphology. Remarkably, the finely engineered NiS*_x_* catalyst synthesized via thermal‐evolution for 24 h (NiS*_x_*‐24 h) exhibits an exceptionally low cell voltage of 1.59 V (lower than Pt/C‐IrO_2_ catalytic couple) for neutral water splitting, which represents the lowest value ever reported. The enhanced performance of NiS*_x_*‐24 h is a multi‐synergized consequence of the simultaneous enrichment of oxygen and hydrogen evolution reaction catalyzing species, accompanied by an optimum electrocatalytic surface area and intrinsic high conductivity. Overall, this innovative work opens a route to engineering the active material's electronic structure/morphology, demonstrating novel Ni^3+^/*p*S_n_^2−^‐enriched NiS*_x_* catalysts which surpass state‐of‐the‐art materials for neutral water splitting.

## Graphical abstract

Here, the simple and highly scalable preparation of earth‐abundant NiS*_x_* bi‐functional electrocatalyst exhibiting the best ever reported performance for neutral water splitting applications is presented. This remarkably enhanced performance is a multi‐synergized consequence of the method's capability to finely engineer the active material's surface electronic structure and morphology, thus surpassing the reported performance of state‐of‐the‐art materials for neutral water splitting.

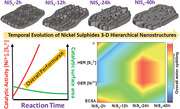

## 1 Introduction

The ever increasing energy demand around the globe requires suitable green energy production alternatives for sustainable development. The cleanest form of energy; hydrogen, is considered a well‐known alternative to the conventional nonrenewable sources, with a high gravimetric energy density.^[^ ^1^ ^]^ Electrochemical generation of hydrogen via water splitting is being massively researched due to its potential for the economic generation of pure hydrogen.^[^ ^2^ ^]^ Even so, water electrolysis still suffers from high overpotentials due to the sluggish oxygen evolution (OER) and hydrogen evolution reactions (HER), thus demands the development of highly active water oxidation/reduction electrocatalysts. Pt and IrO_2_/RuO_2_‐based noble‐metal electrocatalysts are benchmarked for HER and OER, respectively, nevertheless, their enormously high cost hinders their industrial scalability and widespread applicability.^[^ ^3^ ^]^ Various transition metal‐based earth‐abundant electrocatalysts were recently reported in the literature with superior OER^[^ ^4^, ^5^ ^]^ and HER performance^[^ ^6^, ^7^ ^]^ separately, under alkaline and acidic conditions, respectively. Regardless, bifunctional electrocatalysis for overall water splitting still suffers due to the tradeoff of better OER and moderate HER performance in alkaline media, and better HER and only moderate OER performance under acidic conditions.^[^ ^8^, ^9^ ^]^ Only recently, major advancement in the development of transition metal‐based bifunctional electrocatalysts for overall water splitting under alkaline conditions was demonstrated in several reports, with cell voltages of ˂1.5 V at 10 mA cm^−2^.^[^ ^10^, ^11^, ^12^ ^]^ However, water splitting technologies still suffer due to the extreme pH conditions of the highly acidic or alkaline electrolytes required for their optimum electrochemical performance. These corrosive conditions are not environmental friendly, and limit the usage of the various catalytic electrodes and cell components in the electrolyzer due to limited stability issues, thus increasing the final device and process costs.^[^ ^13^ ^]^

To overcome these handicapping limitations, water splitting under neutral conditions is a plausible technological solution, as it can be performed without the requirement of special membranes, expensive catalytic electrodes, and complex cell setups.^[^ ^14^ ^]^ Furthermore, various biological electrolysis systems (e.g., microbial electrolysis cell) can be integrated under the same umbrella, as they require neutral media working conditions for their operation. However, in spite of the tremendous research efforts on the application of transition metal‐based earth‐abundant electrocatalysts, only a scarce amount of reports on water splitting under neutral conditions can be found in the literature. Cobalt‐ and nickel‐based catalytic systems gained major interest in the pioneering research for neutral water‐based electrolytic systems. Xie et al. reported the electrocatalytic oxidation of water in neutral medium using Co_2_P^[^ ^15^ ^]^ and cobalt borate^[^ ^16^ ^]^ with an overpotential of 320 and 290 mV, respectively. Zhang et al.^[^ ^17^ ^]^ and Wang et al.^[^ ^18^ ^]^ explored CoO‐modified CoSe and Co_4_N as bifunctional electrocatalysts for overall water splitting in neutral water, with a cell voltage of 2.18 and 1.79 V at 10 mA cm^−2^, respectively. Chen et al. explored Ni_3_N@Ni‐Bi as a bifunctional water splitting electrode with a cell voltage of 1.95 V at 10 mA cm^−2^.^[^ ^19^ ^]^ Recently, Cho et al. explored the application of high‐entropy CuCoNiFeMn alloys as electrocatalysts under neutral conditions, exhibiting an overpotential of 320 and 680 mV for HER and OER, respectively.^[^ ^20^ ^]^ In an interesting recent work, Sun et al. applied activated LDH with multi‐vacancies by memory effect for efficient water splitting under neutral conditions.^[^ ^21^ ^]^ It can be observed that the highly‐scalable cost‐effective neutral water splitting process using earth‐abundant catalysts is still an immense mostly unmet challenge, as the current state‐of‐the‐art catalytic materials still suffer from overpotentials considerably higher than the theoretical cell voltage of 1.23 V. Furthermore, the synthetic procedures applied in literature reports for creating highly catalytic materials are far from economically and scalability‐wise worthy. Hence, there is an urgent current need for the development of earth‐abundant electrocatalytic electrode materials for the efficient hydrogen production under neutral conditions, that can further be economically scaled up for mass production applications.

In this context, transition metal‐based sulfides are known in the literature for their efficient catalytic properties. Specifically, nickel sulfides have gained special interest in the field of electrocatalysis due to their high intrinsic electrical conductivity, low cost, and environmental benignity.^[^ ^22^, ^23^, ^24^ ^]^ In a recent report, Zeng et al.^[^ ^25^ ^]^ investigated the enhanced OER and HER performance of NiS_2(1−_ *_x_*_)_Se_2_ *_x_* in comparison to NiS_2_. For this purpose, water splitting under neutral conditions was performed using NiS_2(1−_ *_x_*_)_Se_2_ *_x_* as a bifunctional electrocatalyst. The NiS_2(1−_ *_x_*_)_Se_2_ *_x_* electrolyzer exhibited a cell voltage of 1.87 V at 10 mA cm^−2^, as presented in our literature comparison of cell voltage for overall water splitting using various electrocatalysts in neutral medium. It is well known that the morphology and chemical states of catalytic centers at the surface of the catalytic substrates can dramatically affect their electrocatalytic performance. In this work, for the first time, the combination electronic structure engineering and surface morphology tuning of highly catalytic earth‐abundant 3D substrates, by a single‐step temporal thermal‐evolution process, is demonstrated for the optimum and exceptional water splitting performance in neutral medium. Novel 3D hierarchical nickel sulfides‐based nanostructured electrodes, with a controlled varying ratio of NiS and NiS_2_ species are synthesized directly from nickel foil substrates at different transformation times in a single‐step thermal process. A systematic temporal thermal‐evolution of the 3D nanostructured substrate is demonstrated, with a temporal modulation of non‐abundant Ni^3+^ and *p*S_n_^2−^ species at the electrode surface. Conventionally, nickel‐based electrocatalysts are majorly present in the form of Ni^2+^‐species, that oxidizes to Ni^3+^‐species in a peroxidation step during water electrolysis, with Ni^3+^‐species being the catalytically active sites for the electrochemical reaction.^[^ ^26^, ^27^ ^]^ Hence, in spite of starting with the Ni^2+^ as the active species, the electrocatalytic surface engineering for enhancing the Ni^3+^ species concentration at the surface can be applied as a key route for the water splitting enhanced electrochemical performance. This can eliminate the pre‐oxidation overpotential, and thus decrease the cell voltage for water oxidation.^[^ ^26^ ^]^ However, the abundance of Ni^3+^ species is scarce, and is only purely present in some organometallic compounds which possess high catalytic activity.^[^ ^28^ ^]^ Furthermore, the polysulfide (*p*S_n_^2−^) species are known to exhibit an optimum free energy for hydrogen adsorption leading to efficient hydrogen production at the cathode.^[^ ^29^, ^30^ ^]^ Herein, a systematic electronic structure engineering for multi‐synergized high surface area Ni^3+^/*p*S_n_^2−^‐enriched 3D conducting electrodes is performed, which demonstrates the best reported performance as a bifunctional electrocatalyst for overall water splitting in neutral medium. Furthermore, the ease of the production process of these electrocatalytically active 3D binder‐free electrodes makes this synthetic approach economically highly scalable to industrial applications.

## 2 Results and Discussions

**Figure 1:**
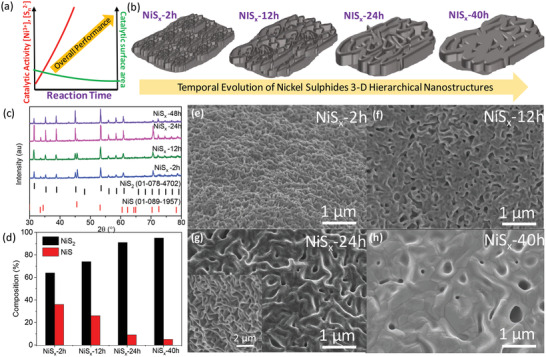
Schematic demonstrating a) the electrocatalytic performance trend and b) thermal evolution of different NiS*_x_* prepared in this study. c) X‐ray Diffraction, d) % composition, and e–h) scanning electron microscopy images of different NiS*_x_* prepared at different transformation times (inset in [g] shows the tilted SEM image).

Overall water splitting urges for an efficient electrocatalyst with high intrinsic electrocatalytic activity and sufficient catalytic surface area for enhanced overall performance. In this work, the temporal thermal‐evolution of nickel sulfides‐based substrates (NiS*_x_*) is performed for engineering the enhanced intrinsic catalytic activity and electrocatalytically active surface area toward overall water splitting under neutral medium (**Figure** **1**a). Furthermore, the electronic states of the Ni metal center and the S anion of our electrocatalysts are successfully engineered and optimized for their water splitting performance. For this purpose, the different NiS*_x_* catalysts are prepared by transforming a Ni foil precursor using a one‐step heating process under an elemental sulfur environment at 450 °C for different transformation times (2, 12, 24, and 40 h, denoted as NiS*_x_*‐2 h, NiS*_x_*‐12 h, NiS*_x_*‐24 h, and NiS*_x_*‐40 h, respectively). It was observed that the different transformation time has a major impact on the electronic environment of the Ni and S atoms along with a significant change in the resulting surface morphology. Since, both the electronic structure and the morphology of the material determine the overall performance of an electrocatalyst, we have successfully optimized the material with synergized contributions of both electronic surface states and morphology.

The measured X‐ray diffraction (XRD) data (Figure 1c) shows the successful transformation of Ni‐foil substrates into mixed phases of hexagonal NiS (01‐089‐1957) and cubic NiS_2_ (01‐078‐4702) with an evident enhancement in the concentration of NiS_2_ phase upon increase in the thermal‐evolution time. NiS*_x_*‐2 h exhibits 64% elemental composition of NiS_2_ which increases to 91% and 95% for NiS*_x_*‐24 h and NiS*_x_*‐40 h, respectively (Figure 1d). Furthermore, the scanning electron microscope (SEM) images depict the successful transformation to hierarchical 3D porous nanostructured substrates for all the samples (Figure 1e–h). Notably, a temporal evolution in the morphology is clearly observed in terms of an increase in the size of surface nanostructures upon increasing the transformation duration time (Figure 1e–h). This can be expected due to densification and coarsening of the grains with increase in the duration of the thermal transformation process. It can be observed that the three‐dimensionality of the material persists at NiS*_x_*‐24 h, which is shown by the tilted SEM image in the inset of Figure 1g. Furthermore, the cross‐sectional SEM image of NiS*_x_*‐24 h shows that the three‐dimensionality of the nanostructures extends to >20 µm depth in the transformed nickel sulfide‐based sample (Figure S1, Supporting Information). It is interesting to observe that all the NiS*_x_* exhibit high conductivity with Ohmic linear *I*–*V* response (Figure S2, Supporting Information). After an initial decrease from NiS*_x_*‐2 h (≈496 S cm^−1^) to NiS*_x_*‐12 h (≈126 S cm^−1^), a systematic increase in the conductivity is observed with value of ≈223 and ≈298 S cm^−1^ for NiS*_x_*‐24 h and NiS*_x_*‐40 h, respectively. The trend in conductivity values is a mutual effect of change in morphology and phase change from NiS to NiS_2_. The initial decrease in the conductivity could be related to the introduction of holes in the NiS*_x_*‐12 h morphology and the systematic increase in conductivity observed henceforth can be due to increase in the concentration of intrinsically high conductivity NiS_2_ from NiS*_x_*‐12 h to NiS*_x_*‐40h.^[^ ^31^ ^]^ These 3D temporally evolved hierarchical nanostructured substrates of intrinsically high‐conducting nickel sulfides can be explored as an electrocatalytically high surface area platform for the electrochemical oxidation and reduction of water under neutral conditions.

**Figure 2:**
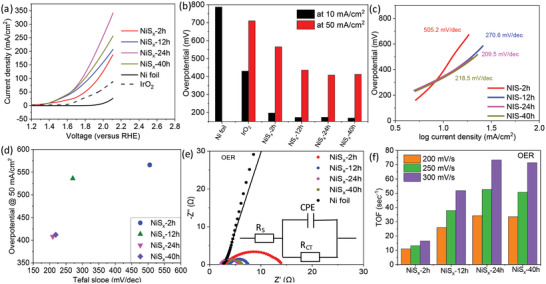
a) Voltage polarization curves, b) overpotential, c) Tafel slope, d) overpotential versus Tafel slope, e) Nyquist plot, and f) TOF toward OER (in neutral conditions) of the nickel sulfide‐based substrates prepared at different transformation times.

The electrocatalytic activity of the different NiS*_x_* substrates was tested as a catalyst for OER under neutral conditions (0.5 m PBS). The voltage polarization curves in the OER region (1.2–2.1 V vs reference hydrogen electrode [RHE]) show that all the nickel sulfide electrocatalysts (NiS*_x_*‐2 h, NiS*_x_*‐12 h, NiS*_x_*‐24 h, and NiS*_x_*‐40 h) exhibit enhanced behavior toward oxidation of water in comparison to the Ni‐foil (**Figure** **2**a, full CV curves are shown in Figure S3, Supporting Information). Interestingly, a systematic increase in the water oxidation performance is observed for the samples prepared at higher duration times till NiS*_x_*‐24 h, with an observed decrease at NiS*_x_*‐40 h (Figure 2b). NiS*_x_*‐24 h shows an ultra‐low overpotential of 173 mV and 408 mV at 10 and 50 mA cm^−2^, respectively. To understand the kinetics of the water oxidation, Tafel slops were analyzed by plotting the overpotential versus log of current density (Figure 2c). In conjugation with the polarization curves, NiS*_x_*‐24 h shows the lowest Tafel slope of 209.5 mV dec^−1^. A clear effect of the temporal evolution of NiS*_x_* nanostructured substrates on the electrochemical water oxidation performance (overpotential and Tafel Slope) can be seen in Figure 2d. Further, the impedance analysis was performed at the OER overpotential, and it was observed that the nickel sulfides‐based substrates exhibit two‐orders lower charge‐transfer resistance in comparison to the Ni‐foil (Figure 2e, inset shows the Randles circuit^[^ ^32^ ^]^). As expected, a systematic decrease in the *R*_CT_ upon increasing transformation time of the nickel sulfides‐based substrates was observed, with NiS*_x_*‐24 h and NiS*_x_*‐40 h exhibiting the lowest *R*_CT_ values of 3.49 and 3.29 Ω, respectively (Figure 2e and Figure S4, Supporting Information). To understand the trend of performance observed for different sulfides‐based substrates, the electrochemically active surface area (ECSA) of all the samples were calculated by evaluating the double layer capacitance (*C*_dl_) in the non‐Faradic region (Figure S5, Supporting Information). Interestingly, NiS*_x_*‐2 h shows the highest ECSA, which decreases with increasing the duration time. This can be related to the hierarchical 3D morphology of NiS*_x_*‐2 h, which further densifies and coarsens at higher duration times, leading to lower ECSA. It is worth noting that in spite of a decrease in ECSA, an enhancement in water oxidation performance is observed from NiS*_x_*‐2 h to NiS*_x_*‐24 h, which decreases at NiS*_x_*‐40 h. To understand this observation, the ECSA‐normalized water oxidation polarization curves were plotted (Figure S6, Supporting Information). NiS*_x_*‐24 h and NiS*_x_*‐40 h show the best performances, in spite of lower ECSA, depicting that the enhanced observed performance of the nickel sulfides‐based substrates prepared at higher duration times is due to enhancement in the intrinsic material catalytic properties. This was further shown by calculating the turn‐over frequency (TOF) of the different substrates. It can be observed that the TOF values increase with the increasing transformation time for the electrode preparation till 24 h (sample: NIS*_x_*‐24 h), and then slightly decrease for NiS*_x_*‐40 h, corresponding well with the ECSA‐normalized water oxidation behavior. Hence, the NiS*_x_*‐24 h shows the best OER performance because of the mutual contribution of enhanced intrinsic catalytic properties and suitable electrocatalytic surface area. However, in NiS*_x_*‐40 h the detrimental effect of the considerable decrease in surface 3D nanostructuring and reduced ECSA decreases the overall OER performance in comparison to NiS*_x_*‐24 h. The chronoamperometric and cyclic voltammetry stability measurements for NiS*_x_*‐24 h were performed in the OER region. NiS*_x_*‐24 h exhibits an insignificant change in its performance after 1000 CV cycles, and 12 h of chronoamperometric measurements for OER (Figure S7, Supporting Information).

**Figure 3:**
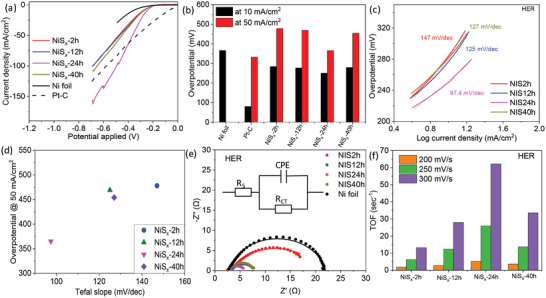
a) Voltage polarization curves, b) overpotential, c) Tafel slope, d) overpotential versus Tafel slope, e) Nyquist plot, and (f) TOF for HER (in neutral conditions) of the nickel sulfides‐based substrates prepared at different transformation times.

The neutral water reduction performance of the NiS*_x_* as a cathode material for HER is evaluated in a three‐electrode electrochemical setup. The voltage polarization curves in the HER region (−0.7 V to 0 V vs RHE) show an enhanced performance of all the NiS*_x_* substrates in comparison to the Ni‐foil (**Figure** **3**a, full CV cycle is shown in Figure S8, Supporting Information). Specifically, NiS*_x_*‐24 h exhibits the highest current density, with the lowest overpotentials of 250 and 365 mV at 10 and 50 mA cm^−2^, respectively (Figure 3b). The Tafel plots show the enhanced kinetics of the NiS*_x_*‐24 h substrate, with the lowest Tafel slope of 97.4 mV dec^−1^ (Figure 3c). Thus, an overall enhancement in the performance of NiS*_x_*‐24 h in terms of overpotential and Tafel slope is clearly observed in Figure 3d. To understand the enhanced performance of NiS*_x_*‐24 h, impedance analysis was performed to calculate the *R*_CT_ (Figure 3e, and Figure S9, Supporting Information). Clearly, a systematic decrease in the *R*_CT_ with increasing the transformation duration time is observed till NiS*_x_*‐24 h, which further decreases at NIS‐40 h. A similar trend was observed in the TOF calculations, with the highest values measured for NiS*_x_*‐24 h (Figure 3f), pointing toward its enhanced intrinsic electrocatalytic activity. To understand the systematic trend in TOF, which otherwise is not observed in the voltage polarization curve (shown in Figure 3a), the ECSA‐normalized voltage polarization curves were plotted (Figure S10, Supporting Information). Interestingly, a similar trend as observed in the TOF calculations is observed, clearly pointing toward the combined role of intrinsic catalytic property and the catalytic surface area in defining the actual overall performance of the material. For NiS*_x_*‐2 h, the intrinsic catalytic activity is moderate; however, the high ECSA gives an actual performance similar to NiS*_x_*‐40 h, which otherwise lacks a large electrocatalytic surface area but exhibits high intrinsic catalytic activity. NiS*_x_*‐24 h, with highest catalytic activity and significant ESCA, exhibits the best HER performance in neutral medium. The chronoamperometric and cyclic voltammetry stability measurements for NiS*_x_*‐24 h were also performed in the HER region. NiS*_x_*‐24 h exhibits an insignificant change in its performance after 1000 CV cycles, and 12 h of chronoamperometric measurements for HER (Figure S11, Supporting Information).

**Figure 4:**
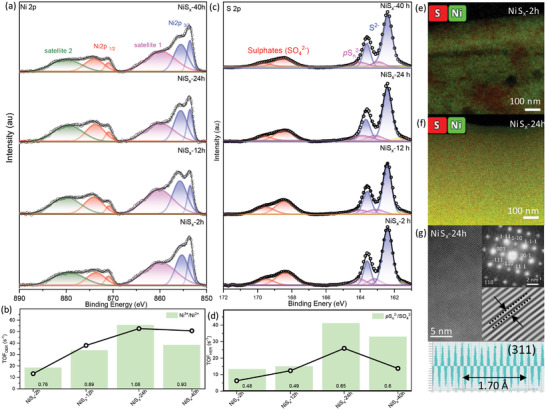
High resolution a) Ni 2p and c) S2p XPS spectra of different NiS*_x_* substrates. Interplay of b) TOF_OER_ values with Ni^3+^:Ni^2+^ and d) TOF_HER_ values with *p*S_n_^2−^/SO_4_^2−^ for different NiS*_x_*. HAADAF sulfur and nickel mapping of e) NiS*_x_*‐2 h and f) NiS*_x_*‐24 h. g) High‐resolution TEM image of NiS*_x_*‐24 h (Inset in [g] shows the selected‐area electron diffraction [SAED] pattern of NiS*_x_*‐24 h).

Undoubtedly, the temporal evolution of morphology and intrinsic catalytic property of the NiS*_x_* substrates has a significant effect on the HER and OER performances under neutral conditions. Hence, it is imperative to get further insight on the chemical state of the catalytic surface of the different nickel sulfides‐based substrates, which majorly dominates their electrocatalytic performance. Ni^3+^ is considered as the active OER species due to the favorable t_2g_^6^e_g_^1^ electronic configuration and improved binding affinity of the Ni^3+^‐enriched catalyst surface for OH^−^ by upshifting the valence band maximum and creating a new unoccupied (hole) state located ≈1 eV above the Fermi level^[^ ^33^, ^34^ ^]^ (a tabulation for various experimental and theoretical reports where Ni^3+^ centers are proved to be the main active species for OER is shown in Table S1, Supporting Information). OER electrode by upshifting the valance band maximum and creating a new unoccupied (hole) state located ≈1 eV above the Fermi level.^[^ ^33^, ^34^ ^]^ Hence, an enrichment of Ni^3+^ centers at the catalyst surface positively influences the resulting OER performance. For this purpose, the Ni^3+^:Ni^2+^ ratio at the catalysts surface was analyzed. The Ni^3+^:Ni^2+^ ratio for different samples can be calculated using the high resolution Ni 2p X‐ray photoelectron spectra (XPS). The high resolution Ni2p XPS spectra for all the sulfides‐based samples exhibit the characteristic Ni 2p_3/2_ and Ni 2p_1/2_ peaks along with their corresponding satellite bands (**Figure** **4**a). The Ni 2p_3/2_ and Ni 2p_1/2_ peaks are further individually split into two peaks, because of the charge transfer from non‐nearest ligands that cause a non‐local screening of the core hole.^[^ ^35^, ^36^ ^]^ The satellite peak in the Ni 2p spectra is assigned to the Ni atoms with unscreened hole in the core level. It is to be noted that Ni^3+^, with d^7^ electrons, has a low spin configuration and does not allow multiplet splitting, whereas, Ni^2+^ with a high spin d^8^ configuration is the sole contributor to the satellite peak.^[^ ^35^ ^]^ For this reason, the ratio of Ni 2p_3/2_:satellite1 can be directly related to the Ni^3+^:Ni^2+^ in any sample. It is very interesting to notice that an increase in the Ni^3+^:Ni^2+^ ratio is observed upon increasing the sample transformation time (Table S2, Supporting Information). The highest Ni^3+^:Ni^2+^ value of 1.08 was observed for NIS*_x_*‐24 h at a transformation time of 24 h, which decreases to 0.93 upon further increasing the transformation time to 40 h. With the increase in the transformation time, the concentration of the OER‐active Ni^3+^ species at the catalyst surface increases due to the oxidation of Ni^2+^ centers. Ni^3+^ is known to be highly electrocatalytically active,^[^ ^26^ ^]^ however, the abundance of Ni^3+^ is limited due to more stable (hence abundant) oxidation state of Ni^2+^. In spite, a twofold increase in the relative concentration of Ni^3+^ in NiS*_x_*‐24 h is observed in comparison to NiS*_x_*‐2 h which is noteworthy. This measured increase in the Ni^3+^:Ni^2+^ ratio is in correlation with the TOF_OER_ values, which show a similar trend of highest TOF value for NIS*_x_*‐24 h, with a slight decrease at NIS*_x_*‐40 h. Figure 4b summarizes the TOF values and the increase in the Ni^3+^:Ni^2+^ ratio following the same trend, thus, sufficiently evidencing the reason for enhancement in the intrinsic OER catalytic activity due to the enriched Ni^3+^ species concentration at the catalyst surface. Furthermore, it is well established that the *p*S_n_^2−^are the highest catalytically active sites for HER, because the H* adsorption energy for the *p*S_n_^2−^ is closer to zero, thus facilitating easy adsorption and desorption of H* during HER^[^ ^29^, ^37^ ^]^ (a tabulation for various experimental and theoretical reports where *p*S_n_^2−^ are proved to be the main active species for HER is shown in Table S4, Supporting Information). On the contrary, SO_4_^2−^ species are the non‐active species at the catalyst surface for the HER. Hence, the *p*S_n_^2−^:SO_4_^2−^ ratio gives the ratio of HER active:HER inactive chemical species at the NiS*_x_* catalyst surface, and thus is explored successfully as a criterion to relate the HER performance of the electrocatalyst to the surface S‐atoms chemical state. The deconvoluted high resolution S2p XPS spectra of all the NiS*_x_* (Figure 4c) show the signature doublet of S 2p_3/2_ and S 2p_1/2_ for terminal (S^2−^) and bridged (*p*S_n_^2−^) polysulfide species and lower (≈162.4 and ≈163.6 eV) and higher binding (≈163.1 and ≈164 eV). Additionally, a doublet for the electrocatalytically dormant sulfate (SO_4_^2−^) species is observed at high binding energy.^[^ ^30^ ^]^ The deconvoluted area under the peak and the exact ratios are tabulated in Table S3, Supporting Information. A controlled tuning of the surface polysulfides in comparison to the oxidized sulfate species (*p*S_n_^2−^/SO_4_^2−^) was observed upon temporal evolution of NiS*_x_*. Interestingly, an enhancement in the HER‐active *p*S_n_^2−^ species surface concentration in comparison to the HER‐dormant SO_4_^2−^ is observed with the highest *p*S_n_^2−^/SO_4_^2−^ ratio of 0.65 for NIS*_x_*‐24 h. Additionally, NiS*_x_*‐24 h exhibits the highest S/Ni ratio of 2.9 indicating the sulfur rich catalyst surface due to relative enhancement in *p*S_n_^2−^ species. A clear correlation between the *p*S_n_^2−^/SO_4_^2−^ ratio and the TOF_HER_ can be observed in Figure 4d justifying the enhanced performance of NiS*_x_*‐24 h due to enriched concentration of polysulfide species at the surface. It is to be noticed that the ratios of Ni^3+^/Ni^2+^ for sample NiS*_x_*‐12 h and NiS*_x_*‐40 h are nearly same (0.89 and 0.93, Figure 4b), however, the TOF_OER_ value for NiS*_x_*‐40 h is higher in comparison to NiS*_x_*‐12 h. This can be explained because of more than twofold increase in the measured intrinsic conductivity (which contributes to the TOF value) of NiS*_x_*‐40 h (≈298 S cm^−1^) in comparison to NiS*_x_*‐12 h (≈126 S cm^−1^) (the conductivity trend is shown in Figure S2, Supporting Information). Similarly, the TOF_HER_ and the *p*S_n_^2−^/SO_4_^2−^ ratio exhibit a similar trend, with slight deviation in the extent of perfect correlation amongst them. From these observations it can be concluded that the intrinsic surface catalytic sites of Ni^3+^ and *p*S_n_^2−^ majorly dictate the trend for HER and OER catalytic activity, along with other important parameters such as the catalyst intrinsic conductivity, crystallinity, and specific crystal facets, which also influence the electrocatalytic performance of different NiS*_x_* catalysts prepared in this work. Nevertheless, the dominance of Ni^3+^ and *p*S_n_^2−^ in dictating the overall performance for OER and HER electrocatalysis, respectively, is successfully demonstrated in this work. Furthermore, the high‐angle annular dark‐field (HAADAF) sulfur and nickel mapping of NiS*_x_*‐2 h shows a non‐uniform distribution of nickel and sulfur, with domains of NiS and NiS_2_ (Figure 4e). Contrastingly, NiS*_x_*‐24 h has uniform distribution of NiS_2_ throughout the sample (Figure 4f). The high‐resolution TEM images shows the highly crystalline lattice fringes of 1.70 Å width corresponding to the (311) plane of NiS_2_ (observed as the highest intensity plane in the XRD data) (Figure 4g). Additionally, the selected‐area electron diffraction pattern along zone axis [110] confirms the single crystalline nature of NiS*_x_*‐24 h sample, and the planes can be indexed to NiS_2_ (ICSD# 646342), corroborating well with the XRD analysis (inset, Figure 4g).

**Figure 5:**
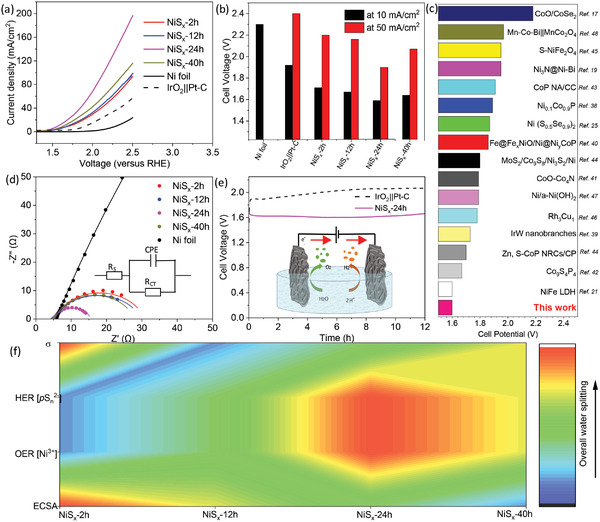
a) Voltage polarization curves. b) Overpotential of the different Ni‐based sulfide samples as bifunctional catalyst for overall water splitting in neutral medium (0.5 m PBS). c) Comparison of cell voltage for overall water splitting using various earth‐abundant electrocatalysts in neutral medium.^[^ ^17^, ^21^, ^25^, ^42^, ^43^, ^44^, ^45^, ^46^, ^47^, ^48^, ^49^, ^50^, ^51^, ^52^ ^]^ d) Nyquist plots for water splitting cells for different NiS*_x_* samples in bifunctional cells. e) Chronoamperometric stability test of NiS*_x_*‐24h||NiS*_x_*‐24 h cell at 10 mA cm^−2^. f) The color‐map profile for the influence of different parameters on overall water splitting performance.

Inspired by the exceptional performance of our NiS*_x_* samples for OER and HER in a three‐electrode setup, a full electrochemical cell for overall water splitting under neutral conditions was assembled. The polarization curves for all the nickel sulfide samples show a steep increase in the current density upon increasing the cell voltage (**Figure** **5**a, full CV cycle is shown in Figure S12, Supporting Information). As expected, the cell voltage at 10 and 50 mA cm^−2^, decreases with increasing the sample transformation time till NiS*_x_*‐24 h, and then increases at NiS*_x_*‐40 h (Figure 5b). NiS*_x_*‐24 h exhibits a cell voltage of 1.59 and 1.94 V at 10 and 50 mA cm^−2^, respectively. To the best of our knowledge, these low cell voltage values have never been reported for earth‐abundant cost‐effective scalable electrocatalysts for water splitting under neutral conditions (Figure 5c). Noteworthy, our cell voltage is considerably lower than the benchmarked IrO_2_||Pt‐C electrode couple. Furthermore, the Nyquist analysis shows the lowest *R*_CT_ value of 10.5 Ω for NiS*_x_*‐24 h, three order of magnitude lower than Ni‐foil (*R*_CT_ = 1005 Ω) and more than twofold lower than that of NiS*_x_*‐2 h (*R*_CT_ = 25.4 Ω) substrates (Figure 5d and Figure S13, Supporting Information). To estimate the Faradic efficiency, the volumes of hydrogen and oxygen evolution for the NiS*_x_*‐24h||NiS*_x_*‐24 h cell were quantified in a homemade electrochemical setup.^[^ ^38^, ^39^ ^]^ The amounts of hydrogen and oxygen evolved during the reaction were in the ratio 2:1, with a Faradic efficiency of 97.7% and 98.1%, respectively (Figure S14, Supporting Information). For practical applicability, stability of the catalyst is one of the important parameters and the chronoamperometric stability test was performed on the NiS*_x_*‐24 h sample. The electrode exhibits an insignificant change in the cell voltage upon continuous operation for 12 h (Figure 5e). The cyclic test for the NiS*_x_*‐24 h||NiS*_x_*‐24 h cell was also performed, and a minimal change in the voltammograms was observed even after 1000 cycles (Figure S15, Supporting Information). The post‐characterization of NiS*_x_*‐24 h from HER and OER electrodes was performed after a 3‐h operation period of the bifunctional cell at 10 mA cm^−2^. The XRD data post‐electrochemical measurements reveals no significant change in the crystal structure, with slight broadening of the XRD peaks probably due to decrease in the crystallinity at the surface (Figure S16a, Supporting Information). High resolution Ni2p XPS analysis shows no change after HER, however a significant shift in the Ni 2p_3/2_ peak position toward high binding energy is observed, which can be related to the formation of the highly electrocatalytic NiOOH/Ni(OH)^[^ ^40^ ^]^ species at the catalyst surface (Figure S16b, Supporting Information).^[^ ^41^ ^]^ The formation of NiOOH/Ni(OH) species at the surface induces a change in the morphology of the post‐OER electrode, and an insignificant change upon HER treatment is observed (Figure S16c,d, Supporting Information). Overall, a methodology for the scalable facile preparation of cost‐effective NiS*_x_*‐based exceptional catalytic electrodes, based on the temporal thermal evolution of the as‐synthesized substrates into hierarchical 3D nanostructured catalysts, is demonstrated in this study. The best catalytic substrate, NiS*_x_*‐24 h, displays a quadruple synergy of enhanced OER active sites (Ni^3+^), HER active sites (*p*S_n_^2−^), ECSA, and conductivity as displayed in Figure 5f, thus acting as an outstanding bifunctional electrocatalyst for overall water splitting under neutral conditions.

Hence, the rational engineering of surface electronic structure and morphology is shown here to deeply affect the catalytic performance of the resulting electrocatalysts. In this work, nickel sulfides with finely engineered electrocatalytically active surface moieties are successfully synthesized, which possess the following advantages over conventional reported nickel sulfides, or any other earth‐abundant electrocatalysts, in general. 1) The methodology developed gives a fine control over the morphology and the electronic structure of the metal center and the anion species by a simple temporal modulation of the process. 2) This is the first report on nickel sulfide as an electrocatalyst where the surface active sites for both OER and HER are observed and analyzed in depth, apart from the general physical and electrochemical characterization usually observed in the literature. 3) The systematic evolution of a highly‐conducting electrocatalytic material is observed, with an enhancement in the surface concentration of electrocatalytic oxygen evolution species (Ni^3+^) and hydrogen evolution species (*p*S_n_^2−^), independently. 4) The optimal electrocatalyst exhibits the lowest cell voltage (1.59 V) as a bifunctional electrocatalyst for overall water splitting in neutral medium ever reported. The measured overpotentials are even lower than the electrochemical cell assembled using the benchmarking precious catalysts IrO_2_ and Pt‐C. 5) The synthesis process is simple, highly economic, and industrially scalable. It is indeed remarkable to observe a finely engineered NiS*_x_* multi‐synergized bifunctional electrocatalyst for exceptional overall water splitting in neutral medium.

## 3 Conclusions

In conclusion, the electronic structure engineered 3D hierarchical nanostructures of highly conducting NiS*_x_* electrocatalysts are synthesized by a single‐step temporal thermal‐evolution of nickel substrates under sulfur environment. With an increase in the evolution time, the catalyst concentration of NiS_2_ increases, accompanied by a controlled tuning of ESCA and conductivity because of structural densification‐induced change in morphology. This temporal evolution of the NiS*_x_*‐based substrates, in turn, induces a significant enhancement in the electrocatalytic water splitting performance under neutral conditions. Sample NiS*_x_*‐24 h, with high intrinsic catalytic activity, significant electrocatalytic surface area, and high conductivity, demonstrates a low overpotential of 173 and 250 mV (at 10 mA cm^−2^) for oxygen and hydrogen evolution, respectively. The two‐electrode bifunctional assembly of the NiS*_x_*‐24h||NiS*_x_*‐24 h electrolyzer exhibits a cell voltage of 1.59 and 1.94 V at 10 and 50 mA cm^−2^, respectively, the lowest reported cell voltage for any earth‐abundant catalytic material applied as a bifunctional electrocatalyst for overall water splitting under neutral conditions. Remarkably, the reported cell voltage is even lower than benchmarked Pt/C‐IrO_2_‐based electrolyzer under neutral conditions. The enhanced performance of the electrocatalyst developed in this work is a synergized contribution of electrocatalytic activity (dominated by the surface electronic states) and the active sites (dominated by the surface morphology). The NiS*_x_*‐24h||NiS*_x_*‐24 h cell shows insignificant performance changes after 12 h of continuous chronoamperometric tests, and after thousand CV cycles. Hence, a methodology for engineering the intrinsic catalytic properties and morphology demonstrated in this study, with the newly developed catalyst sample NiS*_x_*‐24 h exhibiting exceptional electrocatalytic performance, can further be explored in a plethora of other electrochemical applications.

## 4 Experimental Section

### Synthesis of Nickel Sulfide‐Based Electrodes

Nickel foil (99.99%) was sonicated in dilute hydrochloric acid (0.1 m) and further cleaned with isopropyl alcohol and acetone several times before use. Nickel foil was then heated in an elemental sulfur environment, in a pre‐evacuated tubular furnace, at a fixed temperature of 450 °C. The desired temperature was ramped at 10 °C min^−1^ till a temperature of 450 °C was reached, and maintained for different time durations of 2, 12, 24, and 40 h, respectively. Upon completion of the thermal process the furnace was cooled naturally. The sulfur source used in this work was molecular sulfur powder (99.99% Sigma), and the nickel‐to‐sulfur weight ratio was optimized to 2:1. All the reagents used in this work were of analytical grade, and used as purchased without any further purification.

### Characterization

XRD studies were performed to determine the crystal structure of the materials using a Bruker D8 Discover diffractometer. The surface morphology and electron dispersive X‐ray were performed using a high‐resolution SEM (ZEISS Gemini SEM 360). The HAADAF elemental mapping and transmission electron microscopy (Fei Themis Z G3) were performed by cutting a lamella sample from the surface of the material using a Thermo Fisher Helios 5 UC focused ion beam (FIB) system, further applied for the analysis of the crystallographic structure and lattice spacing. The surface chemical composition was analyzed using the XPS system, and measurements conducted on an ESCALAB QXi X‐ray Photoelectron Spectrometer Microprobe. The XPS data was fitted using the Fityk software.

### Electrochemical Measurements

All the electrochemical measurements were conducted by a Metrohm Autolab potentiostat. The three‐electrode measurements were conducted using a platinum mesh as counter electrode and Ag/AgCl as reference electrode, in pH 7 potassium phosphate buffer (0.5 m) as electrolyte. All the potentials were converted to the RHE, and were presented without performing any iR corrections. A working area of 0.5 cm^2^ was fixed for the working electrode. The cyclic voltammetry measurements were conducted at a scan rate of 5 mV s^−1^ in the OER and HER regions, and the reverse scan was considered for calculating the overpotential values. The ECSA was calculated using the formula, ECSA = $\frac{C_{\mathrm{dl}}}{C_{s}}$ cm^2^_ESCA_, where, *C*_s_ = 40 µF cm^−2^ (specific capacitance of flat surface).^[^ ^12^ ^]^ The TOF was calculated using the formula TOF = $\frac{1}{2\mathrm{nF}}$ ^[^ ^12^ ^]^ where, *F*: Faraday constant, and *n*: number of active sites; *n* = *Q*/2*F*, calculated by performing CV in the non‐Faradic region. Nyquist analysis was performed in the frequency range 0.1–10^6^ Hz at the overpotential in the OER and HER region and the plots were fitted using the EIS spectrum analyzer. The activity of the NiS*_x_* electrocatalysts was compared with the commercial IrO_2_ (99% Angene Chemicals Pvt. Ltd.) for OER, Pt‐C (10 wt% Pt on carbon, Sigma Aldrich) for HER and IrO_2_||Pt‐C for full cell. For electrode preparation, 10 mL dispersion of active material was prepared via sonication in a water‐ethanol (4:1) mixture with 50 µL Nafion (of 5%) in ethanol. The electrodes were prepared by drop‐casting the dispersion of active material on Ni foam with a mass loading of ≈1 mg cm^−2^.

## Conflict of Interest

The authors declare no conflict of interest.

## Supporting information

Supporting Information

## Data Availability

The data that support the findings of this study are available from the corresponding author upon reasonable request.
