# Supplementary material for: Electronic Structure Engineering of Highly‐Scalable Earth‐Abundant Multi‐Synergized Electrocatalyst for Exceptional Overall Water Splitting in Neutral Medium
Source: Adv Sci (Weinh). 2022 Nov 11;9(36):2203678. doi: 10.1002/advs.202203678 (PMC9798964; doi:10.1002/advs.202203678)
Supplement: Supplementary file 1 — Supporting Information [file ADVS-9-2203678-s001.pdf]

ADVANCED SCIENCE
Open Access

Supporting Information

for *Adv. Sci.*, DOI 10.1002/advs.202203678

Electronic Structure Engineering of Highly-Scalable Earth-Abundant Multi-Synergized Electrocatalyst for Exceptional Overall Water Splitting in Neutral Medium

*Gaurav Bahuguna, Adam Cohen, Boris Filanovsky and Fernando Patolsky**

Supporting Information

Electronic Structure Engineering of Highly-Scalable Earth-Abundant Multi-Synergized Electrocatalyst for Exceptional Overall Water Splitting in Neutral Medium

Gaurav Bahuguna[1], Adam Cohen[1,2], Boris Flanovsky[1], Fernando Patolsky[1,2*]

[1]*School of Chemistry, Faculty of Exact Sciences, Tel Aviv University, Tel Aviv, 69978, Israel.*

[2]*Department of Materials Science and Engineering, the Iby and Aladar Fleischman Faculty of Engineering, Tel Aviv University, Tel Aviv 69978, Israel.*

[*]*Corresponding Author: fernando@post.tau.ac.il*

Table of Content:

**Figure S1**	Cross sectional SEM image of NiS_x-24h.
**Figure S2:**	(a) IV plots and (b) bar graph representation of corresponding conductivity values for different NiS_x
**Figure S3**	Cyclic voltammograms (5 mV/s) of different NiS_x in the oxygen evolution region.
**Figure S4**	Bar graph representation of R_{CT} values calculated for different NiS_x in the oxygen evolution region.
**Figure S5**	ΔJ versus scan rate and (b) ECSA for NiS_x Ni-based sulphides in the non-faradic region. (Note: ECSA = $\frac{C_{dl}}{C_s}$ cm^2_{ESCA}; where, C_s is the specific capacitance of flat surface in 0.5 M PBS (40 µF cm^{-2})).
**Figure S6**	ECSA normalized OER polarization curve of different NiS_x in 0.5 M PBS.
**Figure S7**	Chronoamperometric stability measurement of NiS_x-24h at 10 mA/cm^2 in the OER region. Inset in figure shows the cyclic stability.
**Figure S8**	Cyclic voltammograms (5 mV/s) of different NiS_x in the hydrogen evolution region.
**Figure S9**	Bar graph representation of R_{CT} values calculated for different NiS_x in the hydrogen evolution region.
**Figure S10**	ECSA normalized HER polarization curve of different NiS_x in 0.5 M PBS.
**Figure S11**	Chronoamperometric stability measurement of NiS_x-24h at -10 mA/cm^2 in the HER region. Inset in figure shows the cyclic stability.
**Table S1**	Tabulation of theoretical and experimental reports revealing Ni^{3+} as the active moiety for catalyzing OER.
**Table S2**	$Ni2p_{3/2}$ and satellite 1 area under the curve and relative Ni^{3+}/Ni^{2+} ratio for different NiS_x samples.
**Table S3**	S_n^{2-} and SO_4^{2-} area under the curve and relative $S_n^{2-}/\ SO_4^{2-}$ ratio for different NiS_x samples.
**Table S4**	Tabulation of theoretical and experimental reports revealing polysulphide (S_n^{2-}) as the active moiety for catalyzing HER.
**Figure S12**	Cyclic voltammograms (5 mV/s) of different NiS_x in as bifunctional catalyst for overall water splitting in 0.5 M PBS.
**Figure S13**	Bar graph representation of R_{CT} values calculated for different NiS_x as bifunctional catalyst for overall water splitting in 0.5 M PBS.

**Figure S14**	Theoretical and experimental yields of hydrogen and oxygen towards overall water splitting using NiS_x-24h.
**Figure S15**	1st and 1000th cyclic voltammetry plot for NiS_x-24h.
**Figure S16**	(a) XRD analysis and (b) High resolution Ni $2p_{3/2}$ XPS analysis and (c,d) SEM images of NiS_x-24h after operation as a bifunctional (post OER and post HER) water splitting electrode in 0.5 M PBS.

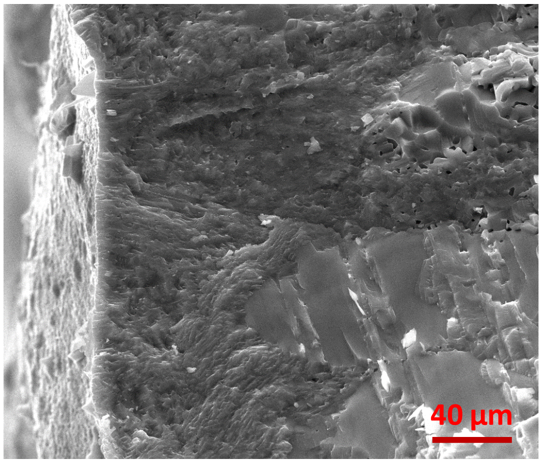

Figure S1: Cross sectional SEM image of NiS_x-24h.

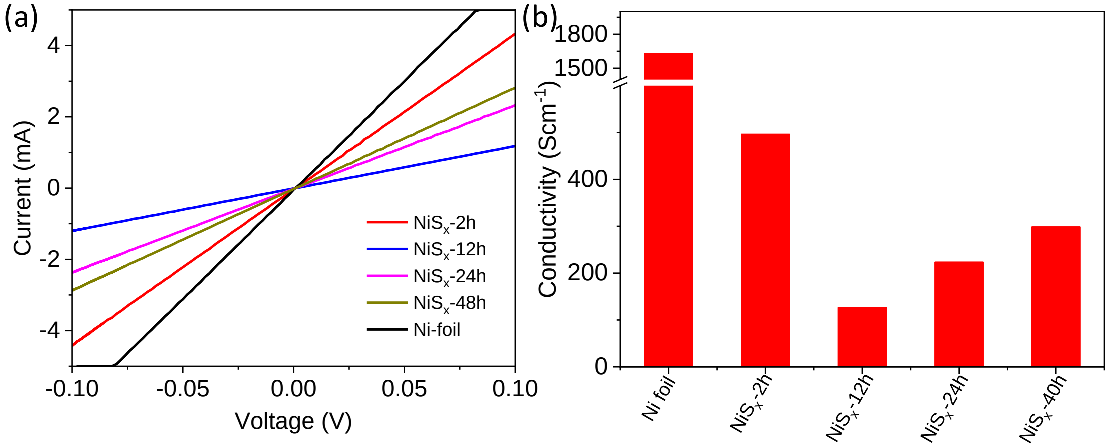

Figure S2: (a) IV plots and (b) bar graph representation of corresponding conductivity values for different NiS_x.

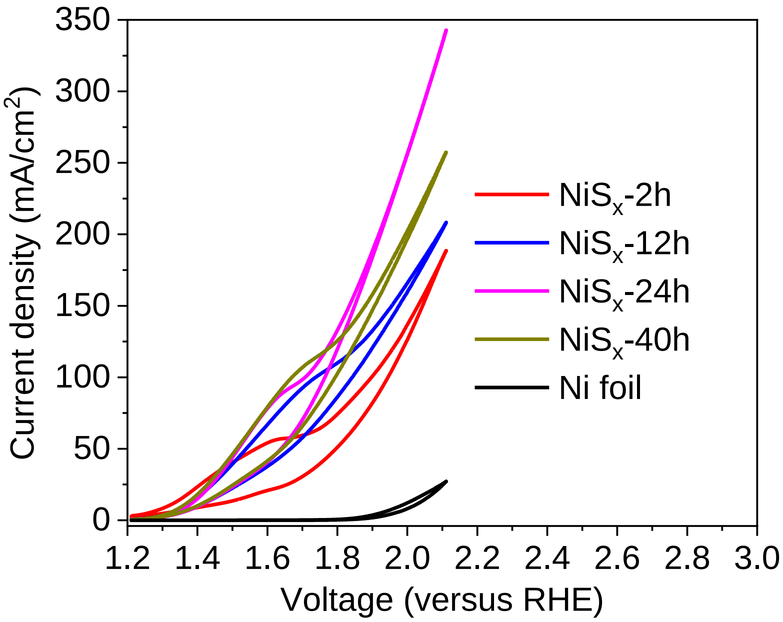

Figure S3: Cyclic voltammograms (5 mV/s) of different NiS_x in the oxygen evolution region.

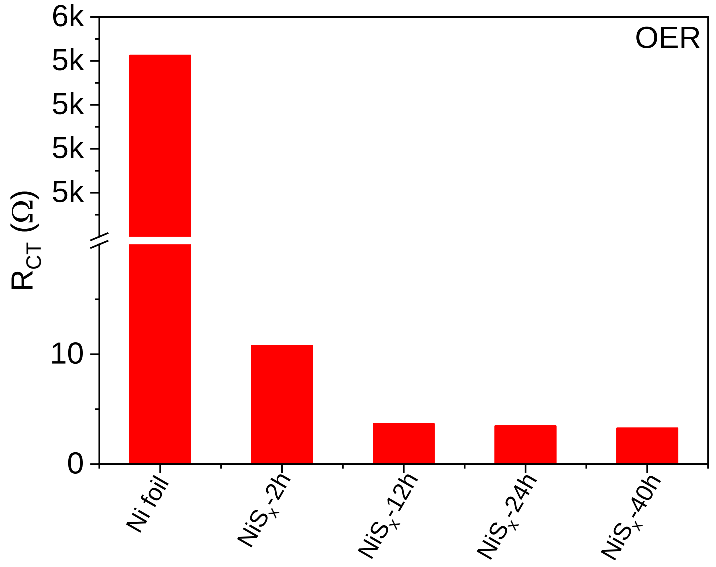

Figure S4: Bar graph representation of R_{CT} values calculated for different NiS_x in the oxygen evolution region.

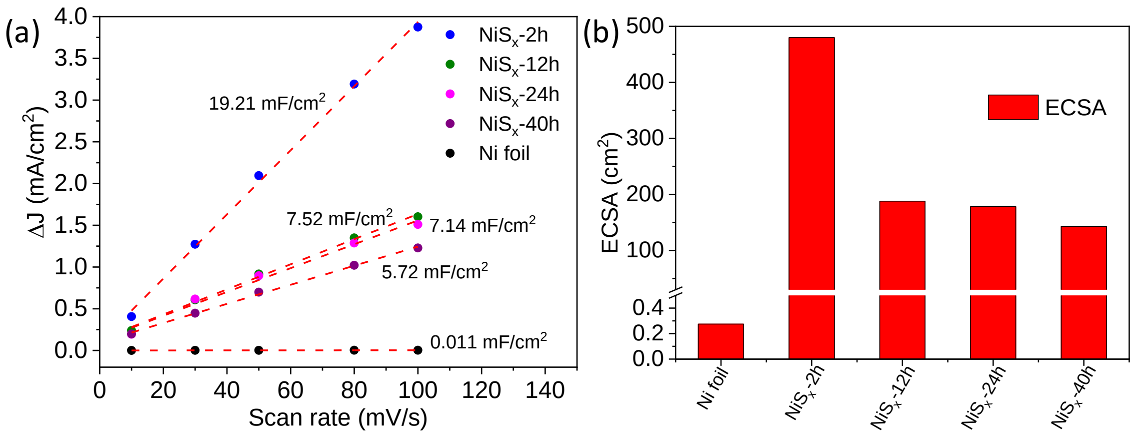

Figure S5: (a) ΔJ versus scan rate and (b) ECSA for NiS_x Ni-based sulphides in the non-faradic region. (Note: ECSA = $\frac{C_{dl}}{C_s}$ $cm^2{}_{ESCA}$; where, C_s is the specific capacitance of flat surface in 0.5 M PBS (40 μF cm^{-2}).

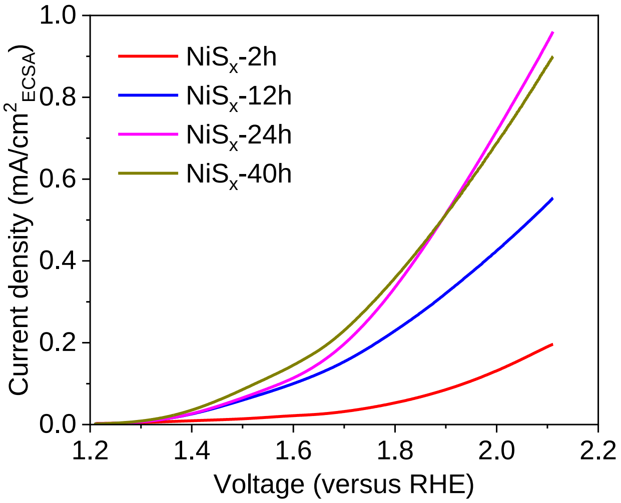

Figure S6: ECSA normalized OER polarization curve of different NiS_x in 0.5 M PBS.

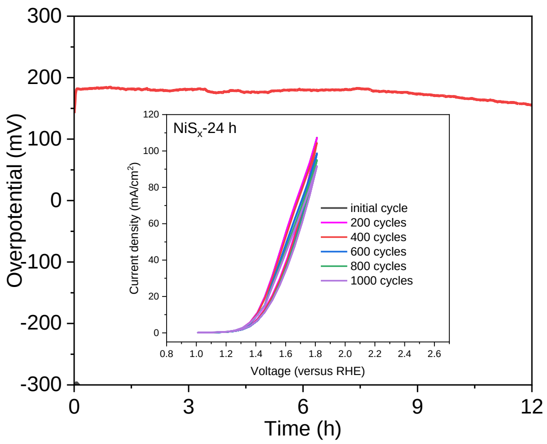

Figure S7: Chronoamperometric stability measurement of NiS$_x$-24h at 10 mA/cm^2 in the OER region. Inset in figure shows the cyclic stability.

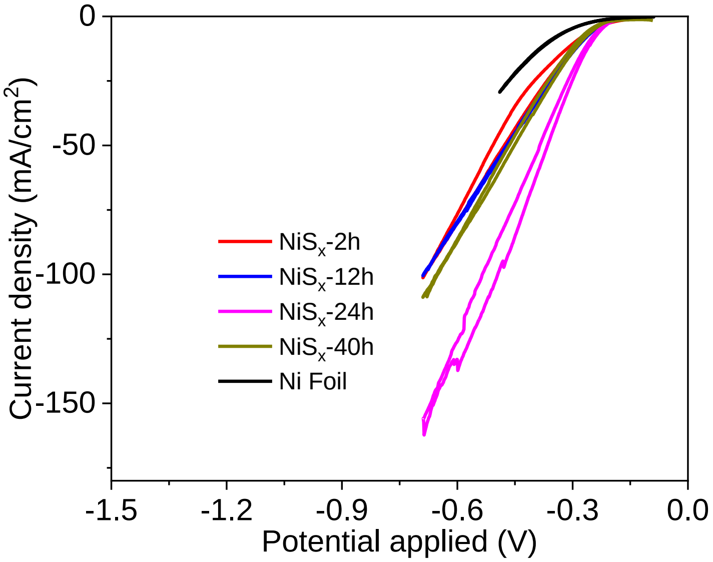

Figure S8: Cyclic voltammograms (5 mV/s) of different NiS$_x$ in the hydrogen evolution region.

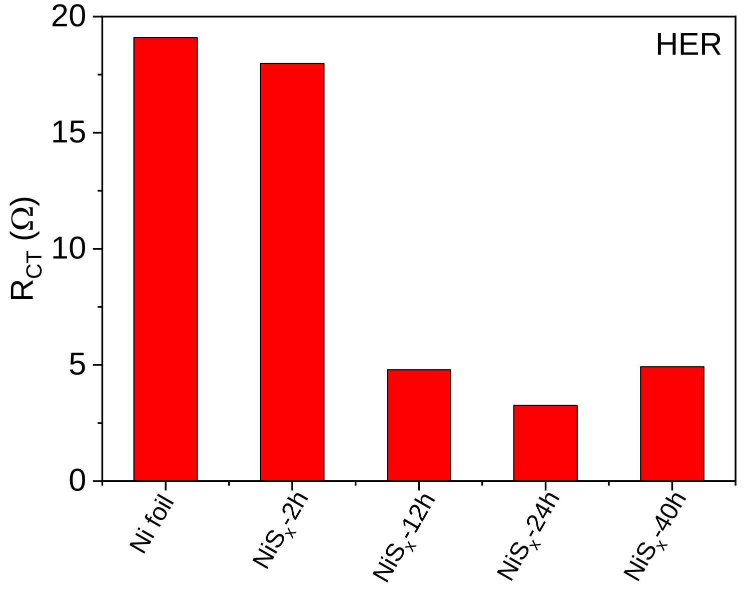

Figure S9: Bar graph representation of R_{CT} values calculated for different NiS_x in the hydrogen evolution region.

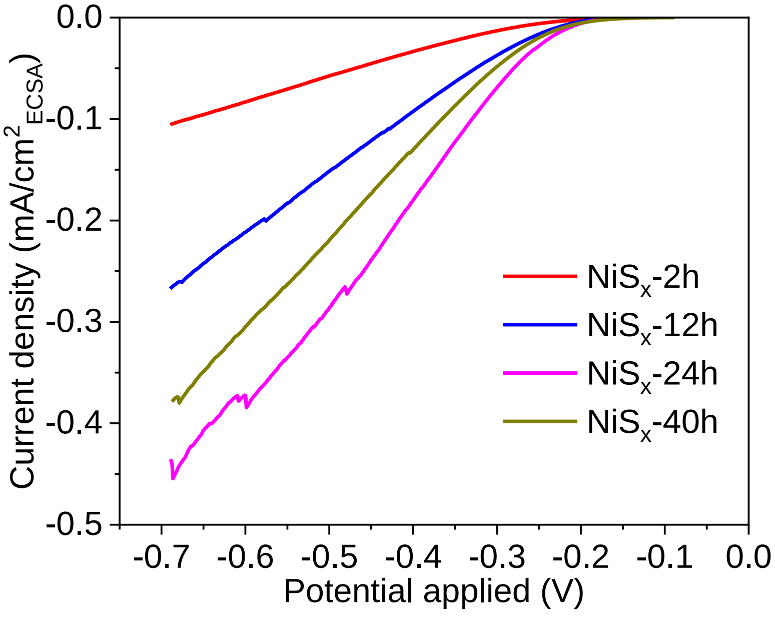

Figure S10: ECSA normalized HER polarization curve of different NiS_x in 0.5 M PBS.

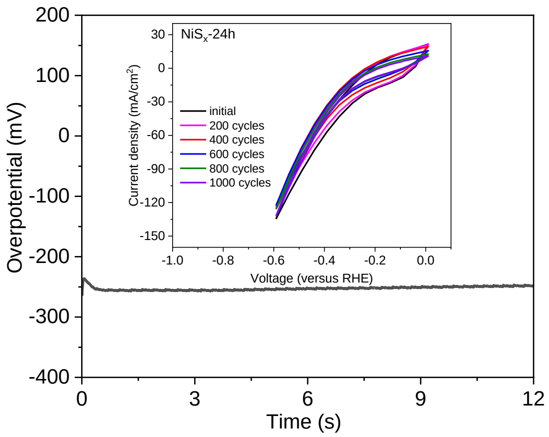

Figure S11: Chronoamperometric stability measurement of NiS_x-24h at -10 mA/cm^2 in the HER region. Inset in figure shows the cyclic stability.

Table S1: Tabulation of theoretical and experimental reports revealing Ni^{3+} as the active moiety for catalyzing OER.

SR no.	Electrocatalyst	Conclusions	Type of study	Journal	Ref
**1**	$Ni_xCo_{3-x}O_4$	Enhancement in the specific OER activity in $Ni_xCo_{3-x}O_4$ is observed for x =1 which is related to the Ni ions in 3+ state. X-ray spectroscopic study reveals the inclusion OER favorable electronic states upon the formation of Ni^{3+} ions which favor the adsorption of -OH surface intermediates, resulting in enhanced OER performance.	Experimental	*Chem. Mater.* 2019, 31, 7618.	[1]
**2**	P-Doped Iron−Nickel Sulfide Nanosheet	Ni^{3+} containing P9.03%-$(Ni,Fe)_3S_2$/NF exhibit enhanced OER activity due to the stronger binding energy of the absorbed OH^- species on the Ni^{3+} sites than that on Ni^{2+} sites.	Theory and Experimental	*ACS Appl. Mater. Interfaces* 2019, 11, 27667.	[2]
**3**	NiFe layered double hydroxides	The high electrocatalytic activity for was related to enriched Ni^{3+} which has an appropriated orbital distribution of $t_{2g}^6e_g^1$ in comparison to that of $t_{2g}^6e_g^0$ for Ni^{2+}. The DFT calculations reveals that the adsorption of O* on Ni^{3+} is enhanced (ΔG_{O*}= 3.03 eV), while the adsorption of OH* species is weakened, leading to energetically favorable transformation O* to OH (ΔG_2= 1.56 eV).	Theory and Experimental	*Small* 2018,14,1800136	[3]
**4**	Perovskite Oxide	This pioneer work systematically examined more than 10 transition metal oxides experimentally and theoretically. The work demonstrated that the occupancy of the 3d electron with an e_g symmetry has volcano–	Theory and Experimental	*Science* 2011, 334, 1383.	[4]

		shaped dependence on the intrinsic OER activity the of surface transition metal cations. of The e_g occupancy of 1 for transition metal cation is demonstrated to be the peak OER activity moiety.			
**5**	$Ni_{0.8}Co_{0.1}Fe_{0.1}O_xH_y$	Doping with Fe results in the enhancement Ni^{3+}/Ni^{2+} leading to an increase in the binding energy of the OH^- adsorption, thus resulting in an overall enhancement in the OER performance.	Experimental	*ACS Catal.* 2018, 8, 5621.	[5]
**6**	Ni–Co Oxide	Ni–Co oxide nanosheet exhibits an overpotential of ≈0.34 V for OER in alkaline media. The enhanced activity is related to Ni^{3+} enriched surface which benefits the formation of main redox site (NiOOH) as revealed by in-situ X-ray absorption fine structure spectroscopy and X-ray absorption near edge structure.	Experimental	*Adv. Energy Mater.* 2015, 5, 1500091.	[6]
**7**	$LiNiO_2$	Electronic structure of NiO was tuned via Li doping to enhance the OER activities. A synergistic combination of synchrotron-based photoemission spectroscopy, X-ray absorption spectroscopy, and density functional theory reveals that the Ni^{3+} oxidation states stabilize the adsorption of OH^- on the electrode, hence facilitating the fast kinetics for OER.	Theory and Experimental	*Chem. Mater.* 2019, 31, 419.	[7]

Table S2: $Ni2p_{3/2}$ and satellite 1 area under the curve and relative Ni^{3+}/Ni^{2+} ratio for different NiS_x samples.

sample	area under the curve		Ni^{3+}/Ni^{2+}
	$Ni2p_{3/2}$	satellite 1	
NIS-2h	66128	86982.9	0.76
NIS-12h	74100.6	83083.4	0.89
NIS-24h	50244	46572.5	1.08
NIS-40h	98084	105314	0.93

Table S3: S_n^{2-} and SO_4^{2-} area under the curve and relative S_n^{2-}/ SO_4^{2-} ratio for different NiS_x samples.

Sample	area under the curve		
	SO_4^{2-}	S_n^{2-}	S_n^{2-}/ SO_4^{2-}
NiS_x-2h	14525	6996	0.48
NiS_x-12h	14956.3	7401.9	0.49
NiS_x-24h	10966.5	7117.9	0.65
NiS_x-40h	17082.1	10239.3	0.60

Table S4: Tabulation of theoretical and experimental reports revealing polysulphide (S_n^{2-}) as the active moiety for catalyzing HER.

SR no.	Electrocatalyst	Conclusions	Type of study	Journal	Ref
**1**	Amorphous MoS_x	Only bridged polysulphides are involved in HER demonstrated via Operando Raman Spectroscopy and DFT calculations.	Theory and Experimental	*ACS Catal.* 2016, 11, 7790.	[8]
**2**	Ni_3S_2 Nanohorn Arrays	Bridging S_n^{2-} at the coupling interface of Ni_3S_2 and VS_4 acts as the active HER catalyst with an overpotential of 177 mV at 10 mA/cm^2 in alkaline medium	Experimental	*Applied Catalysis B: Environmental* 2019, 257, 117911.	[9]
**3**	Anodically deposited MoS_6	An ability to control bridging S_n^{2-} via precursor choice and electrochemistry a systematically control over electrochemical activity for HER catalysis was attained.	Experimental	*ACS Appl. Mater. Interfaces* 2019, 11, 32879.	[10]
**4**	Dimeric $[Mo_2S_{12}]^{2-}$ Cluster	Bridging polysulphide is reported as the dominant HER catalytic Moiety. Electrocatalyst with enriched polysulphide (S_n^{2-}) species demonstrates lowest overpotential and ΔG_{H*} closer to zero (-0.05eV)	Theory and Experimental	*Angew. Chem* 2015, 15181.	[11]
**5**	$[Mo_3S_{13}]^{2-}$	To distinguish the catalytic performances of apical (S^{2-}) and polysulphide (S_n^{2-}) species,	Theory and Experimental	*ACS Catal.* 2016, 6, 861.	[12]

		density functional theory calculation were performed. The bridged S_n^{2-} species were found to be the HER catalyst with ΔG_{H*} closer to zero in comparison to apical (S^{2-}) species. In situ Raman spectroscopy of the $[Mo_3S_{13}]^{2-}$ further demonstrate the higher catalytic reactivity of the polysulphide (S_n^{2-}) over apical (S^{2-}) for proton reduction.			
**6**	S-rich Cobalt Polysulfide	Excellent HER performance with an overpotential of 42 mV at 10 mA/cm^2 in alkaline medium was attained. The superior performance of the S-rich CoS_x composite was attributed to the unique interconnected silk-cocoon structure and the polysulfide composition.	Experimental	*Energy Environ. Sci* 2018, 11, 2467.	[13]
**7**	Graphene-encapsulated CoNi	A high-performance electrolyzer was designed, using polysulfides as mediators and graphene-encapsulated CoNi as catalysts. It produced H_2 with a low potential of 0.82 V at 100 mA/cm^2.	Experimental and theory	*The Innovation 2*, 2021, 100144.	[14]

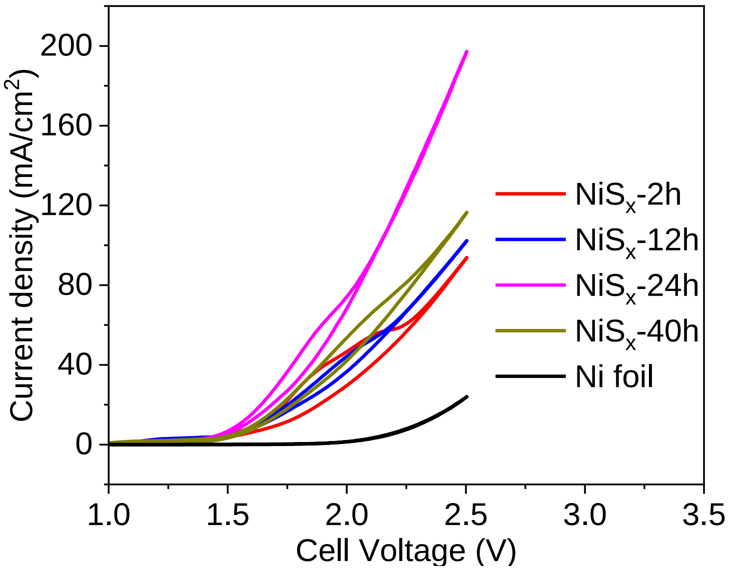

Figure S12: Cyclic voltammograms (5 mV/s) of different NiS_x in as bifunctional catalyst for overall water splitting in 0.5 M PBS.

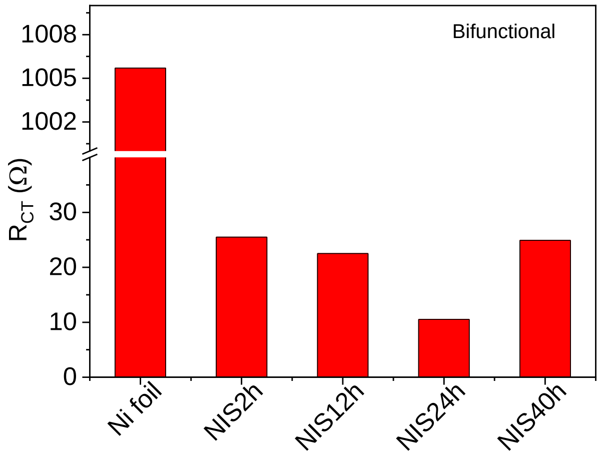

Figure S13: Bar graph representation of R_{CT} values calculated for different NiS_x as bifunctional catalyst for overall water splitting in 0.5 M PBS.

\

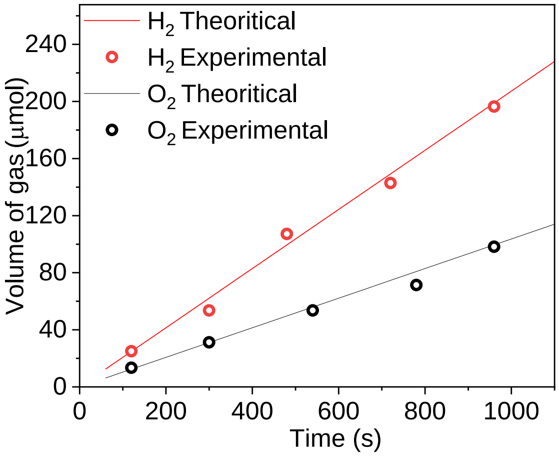

Figure S14: Theoretical and experimental yields of hydrogen and oxygen towards overall water splitting using NiS_x-24h.

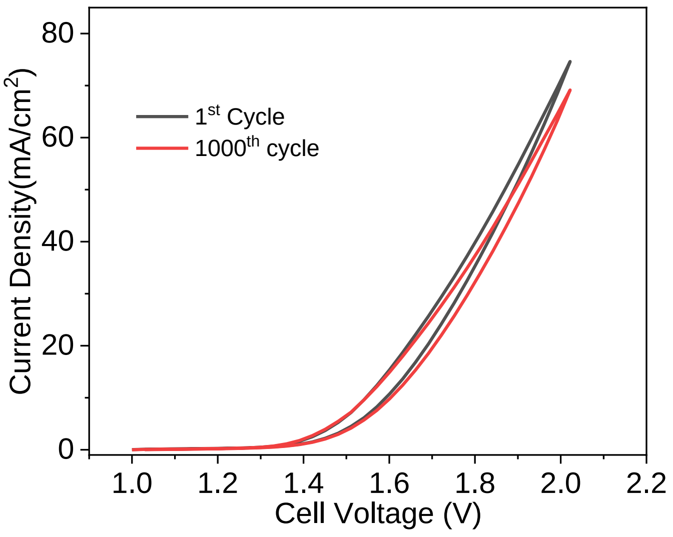

Figure S15: 1st and 1000th cyclic voltammetry plot for NiS_x-24h.

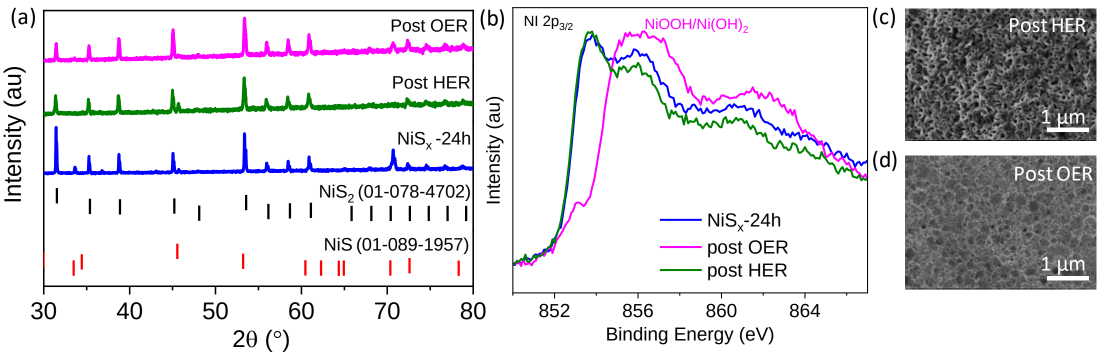

Figure S16: (a) XRD analysis and (b) High resolution Ni $2p_{3/2}$ XPS analysis and (c,d) SEM images of NiS_x-24h after operation as a bifunctional (post OER and post HER) water splitting electrode in 0.5 M PBS.

References:

[1] M. Cui, X. Ding, X. Huang, Z. Shen, T. L. Lee, F. E. Oropeza, J. P. Hofmann, E. J. M. Hensen, K. H. L. Zhang, *Chem. Mater.* **2019**, *31*, 7618.

[2] C. Liu, D. Jia, Q. Hao, X. Zheng, Y. Li, C. Tang, H. Liu, J. Zhang, X. Zheng, *ACS Appl. Mater. Interfaces* **2019**, *11*, 27667.

[3] Y. Wang, M. Qiao, Y. Li, S. Wang, *Small* **2018**, *14*, 1.

[4] J. Suntivich, K. J. May, H. A. Gasteiger, J. B. Goodenough, Y. Shao-horn, F. Calle-vallejo, A. D. Oscar, M. J. Kolb, M. T. M. Koper, J. Suntivich, K. J. May, H. A. Gasteiger, J. B. Goodenough, Y. Shao-horn, *Science (80-.).* **2011**, *334*, 2010.

[5] Q. Zhao, J. Yang, M. Liu, R. Wang, G. Zhang, H. Wang, H. Tang, C. Liu, Z. Mei, H. Chen, F. Pan, *ACS Catal.* **2018**, *8*, 5621.

[6] H. Y. Wang, Y. Y. Hsu, R. Chen, T. S. Chan, H. M. Chen, B. Liu, *Adv. Energy Mater.* **2015**, *5*, 1.

[7] G. Fu, X. Wen, S. Xi, Z. Chen, W. Li, J. Y. Zhang, A. Tadich, R. Wu, D. C. Qi, Y. Du, J. Cheng, K. H. L. Zhang, *Chem. Mater.* **2019**, *31*, 419.

[8] Y. Deng, L. R. L. Ting, P. H. L. Neo, Y. J. Zhang, A. A. Peterson, B. S. Yeo, *ACS Catal.* **2016**, *6*, 7790.

[9] D. Yang, L. Cao, L. Feng, J. Huang, K. Kajiyoshi, Y. Feng, Q. Liu, W. Li, L. Feng, G. Hai, *Appl. Catal. B Environ.* **2019**, *257*, 117911.

[10] O. Mabayoje, Y. Liu, M. Wang, A. Shoola, A. M. Ebrahim, A. I. Frenkel, C. B. Mullins, *ACS Appl. Mater. Interfaces* **2019**, *11*, 32879.

[11] Z. Huang, W. Luo, L. Ma, M. Yu, X. Ren, M. He, S. Polen, K. Click, B. Garrett, J. Lu, K. Amine, C. Hadad, W. Chen, A. Asthagiri, Y. Wu, *Angew. Chemie - Int. Ed.* **2015**, *54*, 15181.

[12] L. R. L. Ting, Y. Deng, L. Ma, Y. J. Zhang, A. A. Peterson, B. S. Yeo, *ACS Catal.* **2016**, *6*, 861.

[13] C. Wang, T. Wang, J. Liu, Y. Zhou, D. Yu, J. K. Cheng, F. Han, Q. Li, J. Chen, Y. Huang, *Energy Environ. Sci.* **2018**, *11*, 2467.

[14] M. Zhang, J. Guan, Y. Tu, S. Wang, D. Deng, *Innov.* **2021**, *2*, DOI 10.1016/j.xinn.2021.100144.
